# *Cyprinus carpio* TRIF Participates in the Innate Immune Response by Inducing NF-κB and IFN Activation and Promoting Apoptosis

**DOI:** 10.3389/fimmu.2021.725150

**Published:** 2021-08-24

**Authors:** Rongrong Liu, Xiaoye Liu, Meijiao Song, Yue Qi, Hua Li, Guiwen Yang, Shijuan Shan

**Affiliations:** Shandong Provincial Key Laboratory of Animal Resistance Biology, College of Life Sciences, Shandong Normal University, Jinan, China

**Keywords:** *Cyprinus carpio* L., TRIF, cellular localization, signaling pathway, apoptosis

## Abstract

TRIF, an important adaptor downstream of Toll-like receptor signaling, plays a critical role in the innate immune response. In this study, the full-length coding sequence of TRIF from common carp (*Cyprinus carpio* L.) was cloned and characterized. Bioinformatics analysis showed that common carp TRIF exhibited a conserved TIR domain and had the closest relationship with grass carp TRIF. Expression analysis revealed that TRIF was constitutively expressed in the examined tissues of common carp, with the highest expression in the spleen and the lowest expression in the head kidney, and could be upregulated under *Aeromonas hydrophila* and poly(I:C) stimulation *in vivo* and under poly(I:C), LPS, PGN, flagellin, and Pam3CSK4 stimulation *in vitro*. Laser confocal microscopy showed that common carp TRIF colocalized with the Golgi apparatus. A luciferase reporter assay showed that carp TRIF elicited the activity of ifn-1 and nf-κb through the C-terminal domain. Additionally, crystal violet staining and qPCR assays revealed that carp TRIF inhibited the replication of SVCV in epithelioma papulosum cyprini (EPC) cells. Then, the signaling downstream of carp TRIF was investigated. Coimmunoprecipitation and Western blotting analysis demonstrated that carp TRIF interacted with TBK1 and augmented the expression of TRAF6 and phosphorylation of TBK1. Overexpression of carp TRIF significantly enhanced the expression of interferon-stimulated genes and inflammatory cytokines. Furthermore, flow cytometric (FCM) analysis suggested that carp TRIF induced apoptosis through the activation of caspase-8. In summary, our study indicated that TRIF plays an essential role in the innate immune responses of common carp against bacterial and viral infection.

## Introduction

Innate immunity plays a crucial role as the first line of defense protecting both lower and higher eukaryotes against pathogenic invasion (1). The mechanism of innate immunity relies on a family of proteins characterized by a highly specialized structure often termed pattern recognition receptors (PRRs) that recognize conserved pathogen-associated molecular patterns (PAMPs) or damage-associated molecular patterns (DAMPs) (2). Toll-like receptors (TLRs), an evolutionary ancient family of PRRs, have been extensively studied over the recent decades (3). Upon the recognition of microbes, TLRs immediately recruit and bind distinct Toll/IL-1 recruitment (TIR) domain-containing adaptor molecules in the cytoplasm (4). To date, six adaptors of TLRs have been identified, although the Toll receptor–associated molecule (TRAM) is absent in teleost genomes (5).

Toll-interleukin 1 receptor domain-containing adaptor molecule (TICAM-1, also named TRIF) is the third TIR domain-containing adaptor protein to be described (6). It has been well documented that TRIF is essential for the TLR3- and TLR4-mediated production of type-I IFN and other proinflammatory mediators in mammals (6, 7). Mammalian TRIF consists of an N-terminal proline-rich region (PRR), a highly conserved TIR domain, and a C-terminal domain that harbors a receptor-interacting protein 1 (RIP1) interaction motif (RHIM) (8). TNF receptor-associated factor (TRAF) 6 and TANK-binding kinase (TBK)-1 interact with TRIF through the N-terminal portion of TRIF, which is crucial for IRF3 and NF-κB activation (7, 9). The TIR domain of TRIF interacts with the TIR domain of TLR3 as well as the TLR4-bridging adaptor TRAM (6, 10). The C-terminal RIP homotypic interaction motif (RHIM) is crucial for NF-κB activation and apoptosis (11–13).

The first sequence of nonmammalian TRIF was found in the catfish genome (14). To date, the complete coding sequence of TRIF has been reported only in the channel catfish (*Ictalurus punctatus*) (15), zebrafish (*Danio rerio*) (16), grass carp (*Ctenopharyngodon idella*) (17), orange-spotted grouper (*Epinephelus coioides*) (18), large yellow croaker (*Larimichthys crocea*) (19), fugu (*Takifugu rubripes*) (20), and black carp (*Mylopharyngodon piceus*) (21). An increasing number of studies have recognized that fish TRIF gene expression can be modulated by bacterial PAMPs, including channel catfish, orange-spotted grouper, and large yellow croaker (15, 18, 19). In other teleost fish, such as zebrafish and grass carp, TRIF can respond to poly(I:C) and grass carp reovirus (GCRV) stimulation (16, 22). In addition, as an adaptor, fish TRIF can execute signal transduction by associating with TBK1 and then phosphorylating IRF3/7, inducing the transcription of IFN-β and other IFN-regulated genes (8). Furthermore, fish TRIF was reported to inhibit virus replication (17, 18). However, the mechanism remains unclear.

Common carp (*Cyprinus carpio* L.) is one of the most important freshwater aquaculture species and an important economic asset for angling and fisheries (23). However, various viruses and bacteria cause serious diseases in common carp, resulting in a high mortality rate and enormous economic losses to the aquaculture industry (24). Therefore, it is imperative to study the immune-related genes of common carp to develop useful treatment strategies for disease prevention and control in common carp (25). In this study, the TRIF gene from common carp was cloned. Its gene expression pattern and immune modulation function were analyzed. In addition, the role of common carp TRIF in the host innate immune responses was studied. These results can lay a foundation for research on the mechanisms of resistance to pathogens and help us to better understand the biological characteristics of TRIF in teleosts.

## Materials and Methods

### Animals and Immune Challenge

Healthy common carp (average weight: 180 g) were purchased from a local fish farm and cultured at 22–25°C in recirculating tap water with commercial fish feed for more than one week prior to experimental use. The immune challenges method and concentration were performed according to the previously described protocols (26). Briefly, fish were injected intraperitoneally with formalin (overnight at 4°C in 0.5% formalin), inactivated *Aeromonas hydrophila* (2 × 10^7^ CFU per fish) and poly(I:C) (1.6 mg/ml) in a volume of 500 μl. Fish in the control group were injected with the same amount of PBS. The samples were collected from three infected fish at different time points after stimulation (0, 3, 6, 12, 24, 72, and 168 h). Tissue samples were stored in liquid nitrogen until subsequent analyses.

### Ribonucleic Acid (RNA) Extraction, Complementary DNA (cDNA) Synthesis, and Real-Time Polymerase Chain Reaction (PCR)

The total RNA of cells and various tissues were extracted using the RNA simple Total RNA Kit (Tiangen, China) according to the instructions of the manufacturer. The total RNA concentration and quality were measured by ultraviolet spectrophotometry (Thermo Fisher Scientific, USA). First-strand cDNA was then synthesized using the FastQuant RT Kit (with gDNase) (Tiangen) following the protocol of the manufacturer. The mRNA expression of target genes was quantified using the SYBR Premix Ex Taq II reagent (Takara, China) and a LightCycler 96 real-time PCR system (Roche, Switzerland). The amplification scheme was as follows: incubation for 30 s at 94°C followed by 40 cycles of 5 s at 94°C, 30 s at 60°C, and 50 s at 70°C. The mRNA expression levels of tissues and cells were normalized to the expression levels of s11 and EF-1α, respectively. The data were analyzed using the 2^−ΔΔCT^ method as described previously (27). Primers are listed in **Supplementary Table 1**.

### Gene Cloning and Plasmid Construction

To obtain the full-length cDNA of the sequence of common carp TRIF, the partial sequence of TRIF was cloned from common carp using a pair of primers specific to the conserved region of the reported TRIF sequence. Then, 5′ and 3′ RACE-PCR were performed using a 3′-full RACE core set (Takara, China) and SMARTer^®^ RACE 5′ Kit (Clontech, USA) according to the instructions of the manufacturer.

To construct the expression of plasmid for GFP-tagged, carp TRIF, or truncated forms including △N (deletion of amino acids 9–158), △TIR domain (deletion of amino acids 324–451), and △C (deletion of amino acids 452–578) were amplified by PCR and inserted into the pEGFP-N1 vector through the appropriate enzyme digestion site to obtain the TRIF-FL-EGFP, TRIF-△N-EGFP, TRIF-△TIR-EGFP, and TRIF-△C-EGFP vectors, respectively. The open reading frames (ORFs) of carp TBK1 was subcloned into the pCMV-HA vector and obtained HA-tagged TBK1 plasmid. For dual-luciferase reporter assays, the 5′ flanking region upstream of the start codon ATG in the *ifn-1* gene was cloned into the PGL4.10 basic plasmid. The generated recombined plasmid was named Luci-*ifn-1*. All constructs were verified by DNA sequencing. The primers used in this study are listed in **Supplementary Table 1**. The luciferase reporter plasmid of *nf-κb* was kindly provided by GuangXun Meng (Institute Pasteur of Shanghai, Chinese Academy of Sciences, China).

### Sequence Analysis

The protein structures of the target genes were predicted by NCBI and SWISS-MODEL. Multiple alignment analysis was performed using Clustal W, and the results were viewed and edited with the BioEdit software. A phylogenetic tree was constructed using the neighbor-joining method and MEGA 6.0 software. To derive the confidence value for the phylogenetic analysis, boot-strap trials were replicated 1,000 times. The GenBank accession numbers for these sequences are shown in **Supplementary Table 2**.

### Isolation and Stimulation of Common Carp Peripheral Blood Leukocytes (PBLs)

Common carp PBLs were prepared by density gradient centrifugation with Percoll (Sigma-Aldrich, Germany) according to a previous protocol (27). Briefly, for the isolation of PBLs, diluted blood was layered on top of 65% Percoll and centrifuged. After 25 min of centrifugation at 800 × g, the cells present on the interface of the gradient were collected and washed three times with PBS. The cells were resuspended in complete L-15 (Gibco, USA) supplemented with 10% fetal bovine serum (Gibco) and 1% penicillin-streptomycin (Gibco). Approximately 10^7^ cells/well were seeded in 24-well plates with 500 μl of complete medium. After recovering overnight at 25°C, drug treatment was performed using poly(I:C) (a synthetic analogue of double-stranded RNA) (5 μg/ml, Sigma-Aldrich), LPS (a component of the outer membranes of gram-negative bacteria) (10 µg/ml, Sigma-Aldrich), peptidoglycan (PGN) (the main PAMP of gram-positive bacteria) (10 μg/ml, Sigma-Aldrich), flagellin (a principal component of bacterial flagella) (10 ng/ml, Sigma-Aldrich), and Pam3CSK4 (a synthetic triacylated lipopeptide that can be recognized by TLR1/2) (10 ng/ml, Invitrogen, USA) at different time points (3, 6, 12, and 24 h) according to the previously described protocols (28). The cells in the control group were stimulated with the same amounts of PBS (denoted by 0 h).

### Cell Culture, Transfections, and Virus

The 293T and HeLa cells maintained in our laboratory were cultured in DMEM (Gibco) supplemented with 10% fetal bovine serum (Gibco) and 1% penicillin-streptomycin (Gibco) in an incubator at 37°C and 5% CO_2_. Epithelioma papulosum cyprini (EPC) cells were cultured at 25°C in M199 medium (Gibco) supplemented with 10% fetal bovine serum (Gibco) and 1% penicillin-streptomycin (Gibco). Lipofectamine 2000 (Invitrogen), Fugene HD (Promega, USA), and jetPRIME reagent (Polyplus, France) were used for 293T, HeLa, and EPC cell transfection, respectively.

Spring viremia of carp virus (SVCV) were kept in the lab and propagated in EPC cells. Viral titers were measured according to the method of Reed and Muench (29). Cytopathic effect (CPE) was observed by crystal violet staining. Briefly, EPC cells were seeded in 24-well plates and transfected with the indicated plasmids. After 24 h, the cells were infected with SVCV for 24 h. The infected EPC cells were washed with PBS and fixed with 4% formaldehyde for 30 min at room temperature, stained with 1% (w/v) crystal violet for 10 min, and then washed with PBS and observed for CPE by a microscope.

### Luciferase Activity Assays

To determine the effects of carp TRIF on the regulation of *nf-κb* and *ifn-1* activity, luciferase assays were performed. 293T cells were seeded in 96-well plates 12 h before transfection. Cells were transfected with Luci-*ifn-1* or Luci-*nf-κb* and pRL-TK (internal control) along with different carp TRIF-overexpressing plasmids. After 24 h of transfection, cells were lysed and analyzed for luciferase activity by Dual-Glo^®^ Reagent (Promega). The relative luciferase activity was calculated by normalizing *Firefly* to *Renilla* luciferase activity. All experiments were performed in triplicate and repeated at least three times.

### Confocal Fluorescence Microscopy

HeLa cells were seeded onto coverslips in 24-well plates. After reaching 70–90% confluence, cells were transfected with TRIF-FL-EGFP, TRIF-△N-EGFP, TRIF-△TIR-EGFP, and TRIF-△C-EGFP for 24 h, respectively. Then, cells on coverslips were washed once with PBS, fixed with 4% paraformaldehyde for 30 min, and stained with WGA (cytomembrane-Tracker) (InvivoGen, France) for 10 min and DAPI (Sigma-Aldrich) for 15 min. For Golgi staining, the cells were washed once with HBSS and stained with Golgi-Tracker (Beyotime, China) at 4°C for 30 min. Finally, the coverslips were washed with PBS and observed by the Leica laser scanning confocal microscope. The results were indicated from three independent replicates.

### Coimmunoprecipitation (Co-IP) and Western Blotting Analysis

For immunoprecipitation (IP) experiments, 3 μg of the indicated plasmids were transfected into 293T cells, which were cultured in 6-well plates overnight. After 48 h of transfection, the medium was removed carefully and washed twice with PBS. Then, the cells were lysed with 600 μl of lysis buffer (1% NP-40, 50 mM Tris-HCl, 150 mM NaCl, 15 mM EDTA, 1 mM NaF, 1 mM Na3VO4, pH 8.0) containing a protease inhibitor cocktail at 4°C for 30 min. The cellular fragments were separated by centrifugation at 12,000 × g for 10 min at 4°C. After centrifugation, the supernatants were transferred into a new centrifuge tube (50 μl of cell lysate was designated as the input). The remaining cell lysates were incubated with protein A/G agarose (Santa Cruz, USA) and monoclonal anti-GFP (Solarbio, China, K106580P, 1:10 diluted with lysis buffer) overnight at 4°C with soft agitation. The agarose-protein complex was harvested and washed three times with a lysis buffer and resuspended in 15 μl of 2 × SDS-PAGE loading buffer (designated as IP). Equal loadings of samples (IP and input) were analyzed by Western blot (WB) with anti-GFP and anti-HA (Abcam, England, ab9110) Abs, respectively.

The generated cell lysates were boiled for 10 min, subjected to 10% SDS-PAGE, and transferred onto PVDF membranes (Sigma-Aldrich). The membranes were blocked with 5% nonfat milk (diluted in TBST buffer; 150 mM NaCl, 3 mM EDTA, 0.1% Tween-20, 50 mM Tris-HCl, pH 8.0) for 1 h at room temperature and incubated with primary antibodies: anti-GFP, anti-TRAF6 (Abcam, ab245319), anti-pTBK1 (CST, USA, D52C2), anti-HA, anti-caspase-8 (CST, 8592S), and anti-β-actin (Solarbio, K200058M) (1:1,000 dilution in blocking milk solution) at 4°C overnight. The membrane was washed three times with TBST and probed with the relevant horseradish peroxidase (HRP)-conjugated secondary antibodies (Proteintech, USA) (1:10,000 dilution in 1% BSA dissolved in TBST) for 1 h at room temperature. Finally, the membrane was washed three times with TBST and processed for ECL Western blotting Detection Reagents (Meilunbio, China). The experiments were performed at least three times.

### Flow Cytometric (FCM) Detection of Apoptotic Cells

Apoptotic cells were evaluated using the Annexin V-mCherry apoptosis detection kit (Beyotime, C1070S). The staining procedure was carried out according to the instructions of the manufacturer. In brief, the EPC cells were seeded in 24-well plates overnight and transfected with pEGFP-N1 or carp TRIF-EGFP. At 24 h post-transfection, cells were harvested, then washed with PBS, and resuspended in Annexin V-mCherry Binding Buffer. Stained cells were incubated with 5 μl of Annexin V-mCherry for 10 min at room temperature in the dark. Then, the stained cells were checked with the FACS Calibur system (BD Biosciences, USA) and evaluated with the FlowJo software (TreeStar, Ashland, OR, USA). The results were calculated from three independent replicates.

### Statistical Analysis

Values are presented as the mean ± SD of three independent experiments with technical replicates for each experiment. Data are processed using one- or two-way ANOVA or Tukey’s test. A value of *P* < 0.05 was considered statistically significant (*P < 0.05, **P < 0.01, ***P < 0.001, **** *P* < 0.0001).

## Results

### Identification of the TIR-Domain-Containing Adapter-Inducing Interferon-β (TRIF) Gene in Common Carp

**Figure 1:**
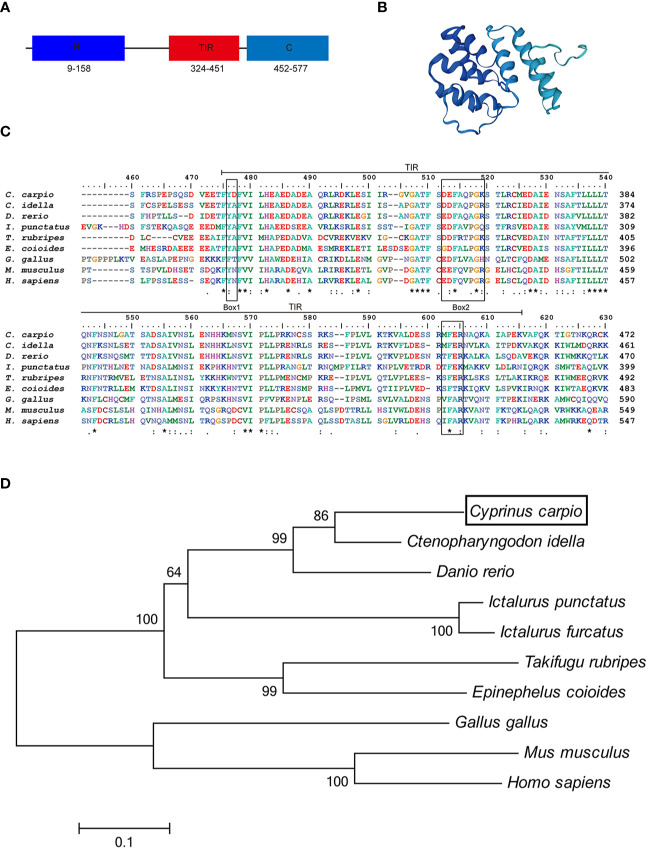
Alignment of TRIF TIR domain sequences and phylogenetic tree. **(A)** A schematic diagram showing the domain architecture of common carp TRIF. **(B)** Modeled three-dimensional structure of common carp TRIF shown as a cartoon. **(C)** The sequences were aligned using the Clustal W method. The identical, conserved, and highly conserved substituted amino acid residues were indicated in (*), (.), and (:) respectively. The three boxes of the TIR domain were outlined in black. **(D)** Phylogenetic analysis of TRIF amino acid sequences. The phylogenetic tree was constructed using amino acid multiple alignments generated by the neighbor-joining method within the MEGA 6.0. program. Black box denoted carp TRIF. The GenBank accession numbers of these sequences are listed in **Supplementary Table 2**. A “*” represents amino acid residues identical among all the nine species in the designated site. One "*" represents amino acid residues identical among all the nine species in the column. "**" or “****” indicates that the amino acid residues of the two or four consecutive sites are identical.

In this study, we cloned and identified a novel TRIF cDNA sequence from common carp (*C. carpio* L.). The complete sequence of the carp TRIF cDNA (GenBank accession No. MZ169546) was 2,667 bp, containing a 5′ untranslated terminal region (UTR) of 343 bp, a 3′ UTR of 590 bp, and an open reading frame (ORF) of 1,734 bp encoding a polypeptide of 578 amino acids (aa) **(** **Supplementary Figure 1** **)**. The protein structure predicted by SWISS-MODEL showed that carp TRIF exhibited the typical characteristics of an N-terminal domain, a TIR domain close to its C-terminus, and a C-terminal domain **(** **Figures 1A, B** **)**. Multiple sequence alignment showed that the TIR domain of TRIF was conserved between common carp and other species, containing three highly conserved regions: Box 1, Box 2, and Box 3. In addition, carp TRIF lacks the N-terminal and C-terminal proline-rich domains **(** **Figure 1C** and **Supplementary Figure 2** **)**.

To examine the evolutionary relationships of TRIF in common carp and other species, a phylogenetic tree was constructed using the neighbor-joining method based on the MEGA 6.0 program. Phylogenetic analysis showed that fish TRIF members formed an independent cluster and carp TRIF had the closest relationship with TRIF in *Ctenopharyngodon idella* and *Danio rerio* **(** **Figure 1D** **)**. In addition, the identities of TRIF between common carp and other teleosts were 22.3–73.1%. Carp TRIF was highly similar to *C. idella* TRIF (73.1%) and had the lowest identity with *Takifugu rubripes* TRIF (30.5%) **(** **Supplementary Table 3** **)**.

### Tissue Expression Profile and Cellular Localization of TIR-Domain-Containing Adapter-Inducing Interferon-β (TRIF)

**Figure 2:**
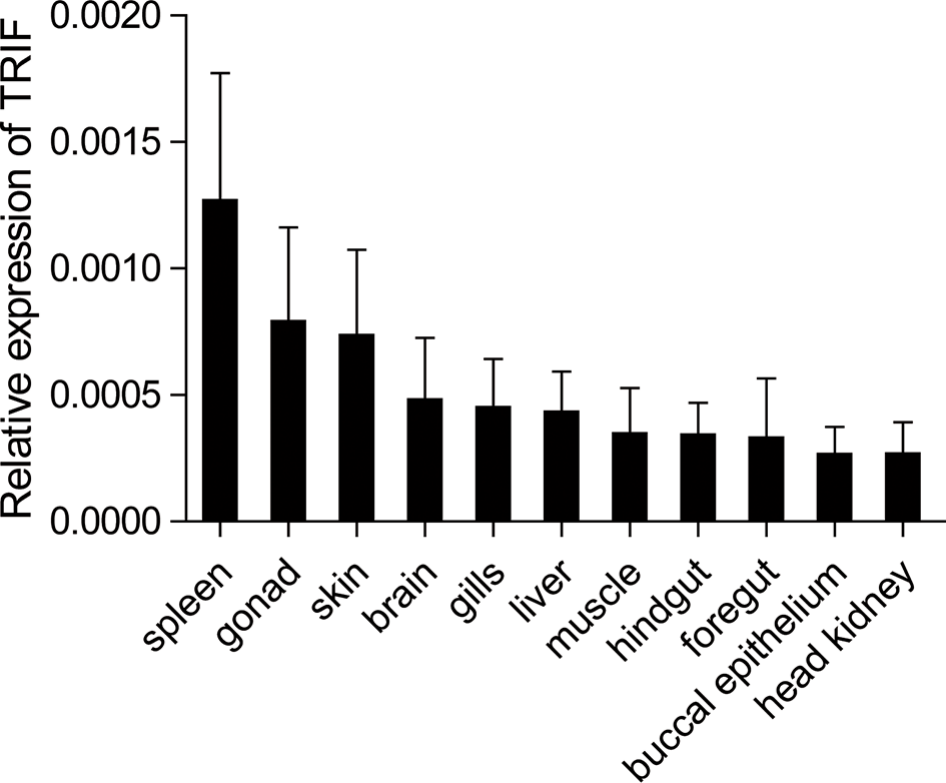
Tissue expression of TRIF in normal common carp. The mRNA expression of carp TRIF in the liver, spleen, head kidney, foregut, hindgut, skin, gills, gonad, muscle, buccal epithelium, and brain was detected by real-time PCR. The 40S ribosomal protein s11 was amplified in each tissue sample as an internal control, n = 3.

The expression patterns of TRIF were detected by qRT-PCR in 11 tissues of healthy common carp, including the liver, spleen, head kidney, foregut, hindgut, gills, gonad, skin, muscle, buccal epithelium, and brain. The results showed that carp TRIF mRNA was detected in all examined tissues, with the highest expression in the spleen, the lowest expression in the head kidney, and moderate expression in the other nine tissues **(** **Figure 2** **)**.

**Figure 3:**
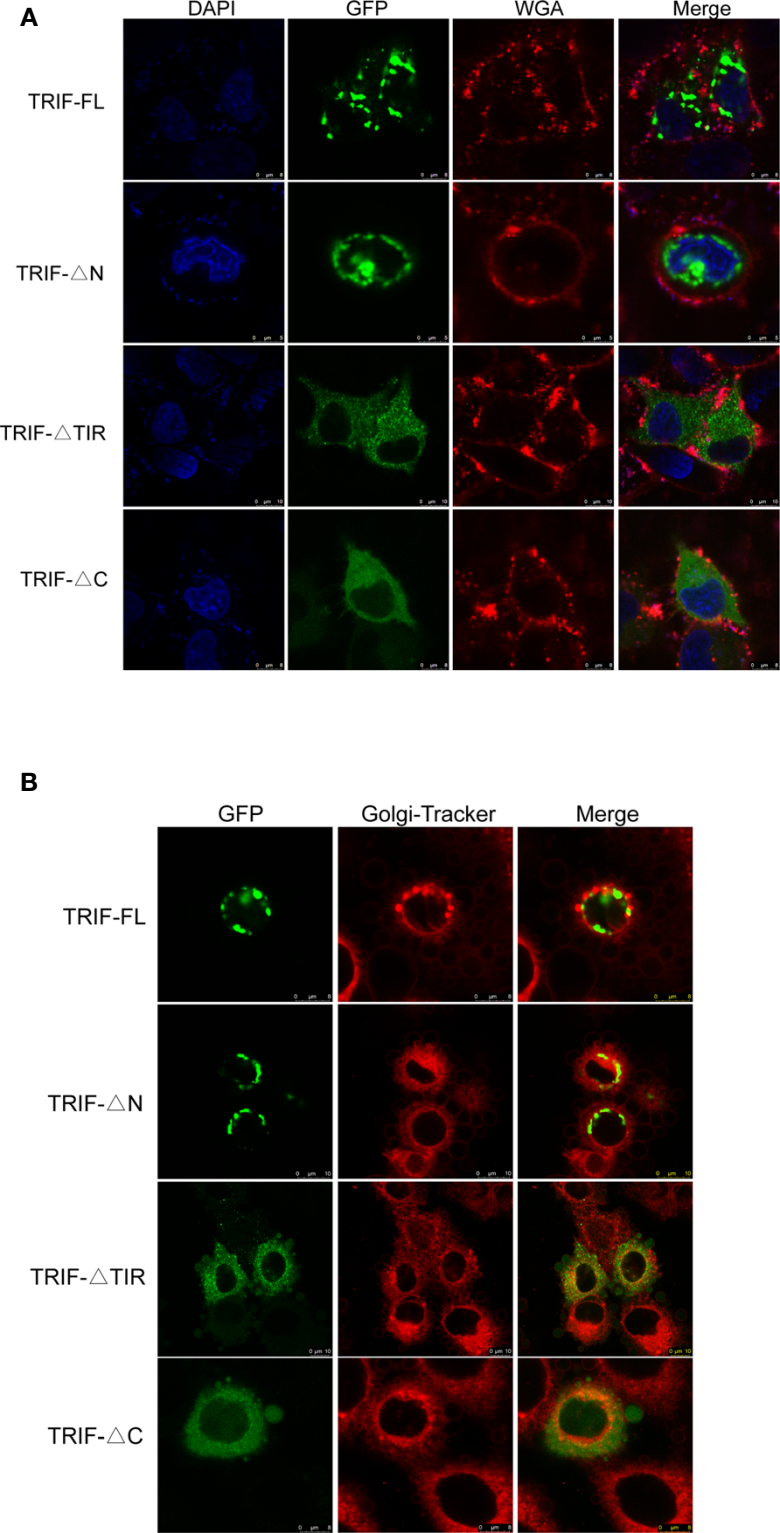
Localization of full-length and truncated forms of carp TRIF. **(A)** Different mutants of carp TRIF, including TRIF-EGFP, TRIF-△N-EGFP, TRIF-△TIR-EGFP, or TRIF-△C-EGFP, were transfected into HeLa cells. At 24 h post-transfection, the cells were stained with WGA (cell membrane marker) and DAPI, then visualized by confocal laser microscopy. Red, green, and blue represented the cell membrane, carp TRIF, and nucleus, respectively. **(B)** HeLa cells were transfected with different mutants of carp TRIF, including TRIF-EGFP, TRIF-△N-EGFP, TRIF-△TIR-EGFP, or TRIF-△C-EGFP. At 24 h post-transfection, the cells were stained with Golgi-Tracker Red and imaged using a laser scanning confocal microscope. Green indicated the TRIF protein. The Golgi complex was stained in red.

To gain a better understanding of the functions of carp TRIF, its subcellular localization was investigated. HeLa cells were transfected with the TRIF-FL-EGFP, TRIF-△N-EGFP, TRIF-△TIR-EGFP, and TRIF-△C-EGFP recombinant vectors and then visualized by confocal microscopy. As illustrated in **Figure 3A**, carp TRIF was localized near the nuclear membrane with the formation of speckle-like structures. The site and structure were likely to be the Golgi apparatus. However, truncated carp TRIF segments had different localizations. The mutant in which the N-terminal domain was deleted showed the same localization as TRIF-FL-EGFP. The two mutants with truncated C-termini or TIR domains were ubiquitously distributed in the cytoplasm. To further investigate whether carp TRIF was accurately localized to the Golgi apparatus, transfection and laser confocal imaging were conducted. The results demonstrated that TRIF-FL and the segment deleting the N-terminal domain were colocalized with the Golgi apparatus, while the two segments with truncated C termini or TIR domains were not similar **(** **Figure 3B** **)**. These results reveal that carp TRIF is a Golgi-localized protein, and the sequence spanning the TIR domain and C-terminus contributes to its unique subcellular localization.

### Expression Profiles of Carp TIR-Domain-Containing Adapter-Inducing Interferon-β (TRIF) After *A. hydrophila* and Poly(I:C) Injection

**Figure 4:**
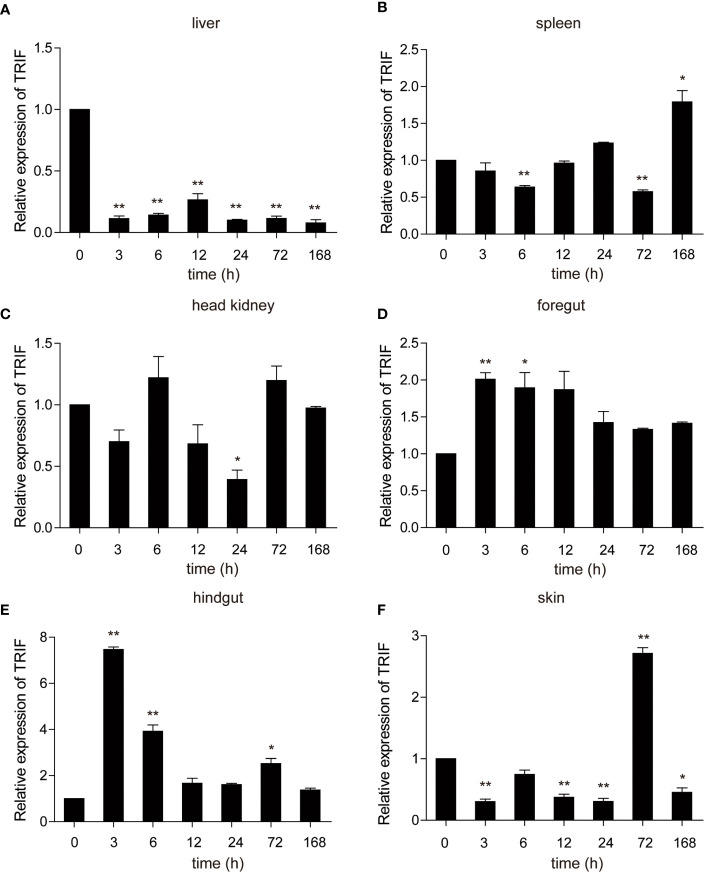
The relative expression of carp TRIF in various tissues of common carp after intraperitoneal injection with *A. hydrophila*. The expression of carp TRIF in the liver **(A)**, spleen **(B)**, head kidney **(C)**, foregut **(D)**, hindgut **(E)**, and skin **(F)** at different time points are shown. The results were calculated relative to the expression of the 40S ribosomal protein s11. Data were presented as a fold increase compared to the unstimulated control group (denoted by 0 h). Means ± SD (n = 3), *P < 0.05, **P < 0.01.

*A. hydrophila*, a well-known fish-pathogenic bacterium, is primarily found in temperate and freshwater environments and causes infections in various organisms (27). The expression profile of carp TRIF was examined in immune-related tissues after bacterial challenge. As illustrated in **Figure 4**, a significant upregulation of TRIF was observed in the spleen, foregut, hindgut, and skin, whereas a downregulation was observed in the liver and head kidney. In the spleen and skin, TRIF expression was initially reduced, then increased and reached a peak at 168 h (1.8-fold) and 72 h (2.7-fold), respectively **(****Figures 4B, F****)**. The expression of TRIF in the foregut and hindgut was significantly elevated and reached maximum levels at 3 h (2.0-fold and 7.5-fold, respectively) **(****Figures 4D, E****)**. However, in the liver and head kidney, the expression of TRIF was downregulated **(****Figures 4A, C****)**. These data suggest that TRIF, as an important adaptor of the TLR signaling pathway, has an important role in antibacterial immune responses.

**Figure 5:**
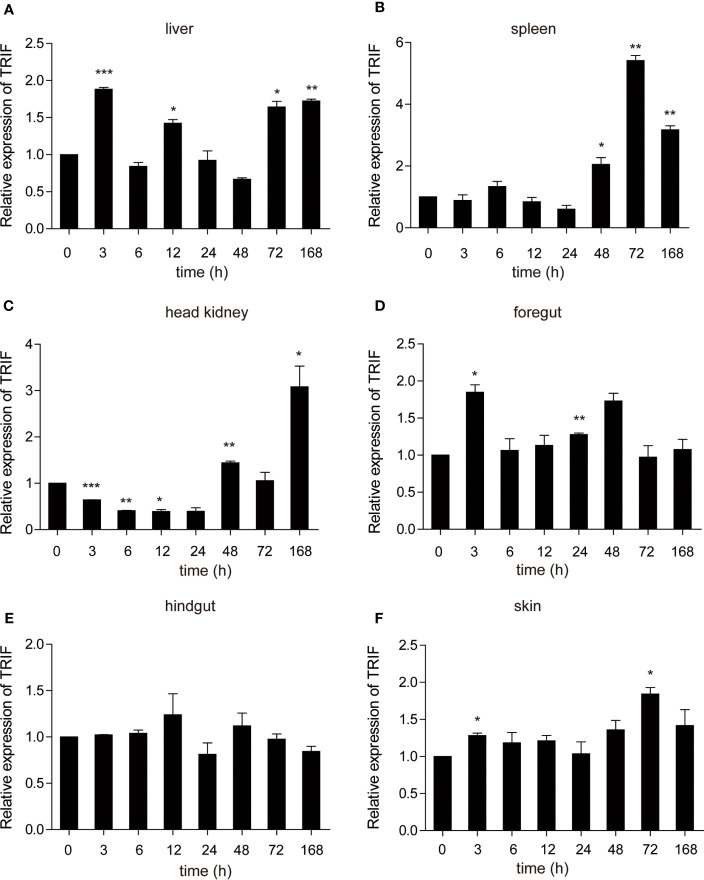
The relative expression of carp TRIF in various tissues of common carp after intraperitoneal injection with poly(I:C). The mRNA expression levels of carp TRIF in the liver **(A)**, spleen **(B)**, head kidney **(C)**, foregut **(D)**, hindgut **(E)**, and skin **(F)** at different time points are shown. Gene expression results were calculated relative to the expression of 40S ribosomal protein s11. Data were presented as the fold changes based on the unstimulated control group (denoted by 0 h). Means ± SD (n = 3), *P < 0.05, **P < 0.01, ***P < 0.001.

Poly (I:C) is a synthetic analogue of double-stranded RNA (dsRNA) that is usually used as a viral infection mimic to induce the immune response (28). The role of carp TRIF in antiviral immunity was investigated. Common carp were injected intraperitoneally with poly(I:C), and the mRNA expression level of carp TRIF was detected at 3, 6, 12, 24, 48, 72, and 168 h post injection. After injection with poly(I:C), a significant upregulation of carp TRIF was observed in the liver, spleen, head kidney, foregut, and skin and reached the highest expression level at different time points (1.9-fold in liver, 5.4-fold in spleen, 3.1-fold in head kidney, 1.8-fold in foregut, and 1.8-fold in skin). No significant differences in carp TRIF expression was observed in the hindgut **(** **Figure 5** **)**.

### Carp TIR-Domain-Containing Adapter-Inducing Interferon-β (TRIF) mRNA Expression in Response to Different Stimuli *In Vitro*

**Figure 6:**
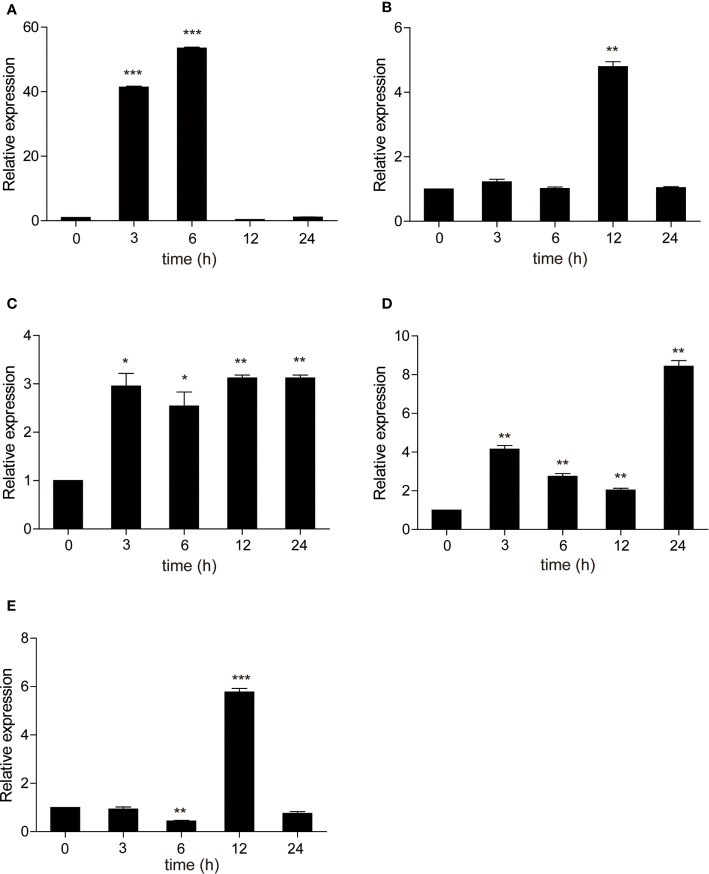
The relative expression of carp TRIF in the PBLs after treatment with poly(I:C) **(A)**, LPS **(B)**, PGN **(C)**, flagellin **(D)**, and Pam3CSK4 **(E)** at different time points. The results were calculated relative to the expression of 40S ribosomal protein s11. Data were presented as the fold change based on the control group (denoted by 0 h). Means ± SD (n = 3), *P < 0.05, **P < 0.01, ***P < 0.001.

As a TLR adapter, it was examined whether carp TRIF gene can respond to TLR ligands. Then, carp TRIF expression was determined after treatment with different immunostimulants in isolated PBLs. As shown in **Figure 6**, the expression of carp TRIF was significantly upregulated at 3 h and peaked at 6 and 12 h (53.54-fold, 3.12-fold) after stimulation with poly(I:C) and PGN, respectively (**Figures 6A, C** **)**. After a challenge with LPS and Pam3CSK4, the expression of carp TRIF increased and reached the highest level at 12 h (4.79-fold and 5.77-fold, respectively) (**Figures 6B, E** **)**. In addition, carp TRIF expression was induced at 3 h and reached a peak value at 24 h (8.43-fold) after flagellin stimulation (**Figure 6D**).

These results indicate that carp TRIF plays a critical role in the immune responses triggered by bacteria and viruses.

### Carp TIR-Domain-Containing Adapter-Inducing Interferon-β (TRIF) Activates IFN and NF-κB Through the C-Terminal Domain

**Figure 7:**
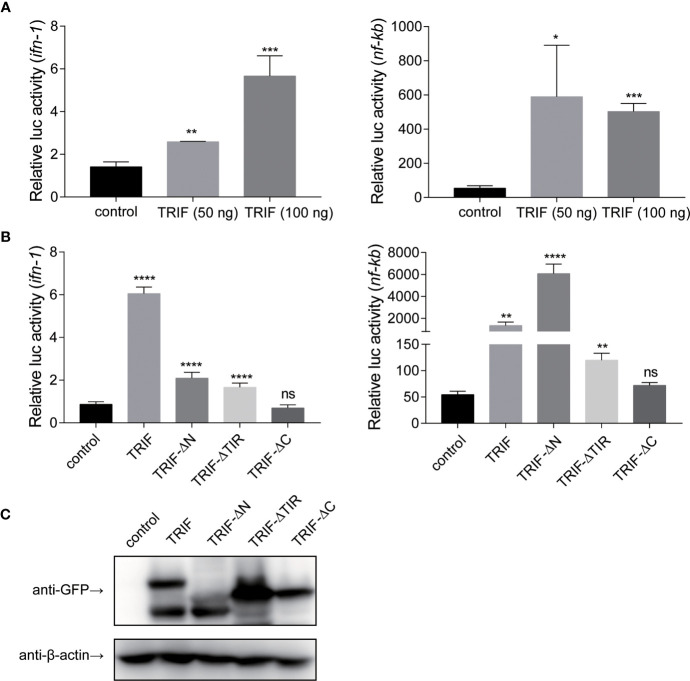
Carp TRIF enhances the promoter activities of *ifn* and *nf-κb* through the C-domain. **(A)** 293T cells were transfected with pEGFP-N1 (control) or different amounts (50 and 100 ng) of carp TRIF-EGFP together with *ifn-1* or *nf-κb* promoter luciferase reporter vector and pRL-TK. After transfection for 48 h, *Firefly* and *Renilla* luciferase activities were measured. **(B)** 293T cells were transfected with the indicated plasmid. After transfection for 48 h, *Firefly* and *Renilla* luciferase activities were measured. **(C)** 293T cells were seeded in 6-well plates and transfected with the pEGFP-N1 (control) and different mutants of carp TRIF (TRIF-△N-EGFP, TRIF-△TIR-EGFP, or TRIF-△C-EGFP) for 48 h. The cell lysates were subjected to Western blot analysis with anti-GFP and anti-β-actin Abs. All data represented the means ± SD (n = 3) from three independent triplicate experiments, *P < 0.05, **P < 0.01, ***P < 0.001, ****P < 0.0001 and ns, no significant.

To further elucidate the mechanisms by which carp TRIF induces an inflammatory response, a luciferase reporter assay was used to test the promoter activities of *ifn-1* and *nf-κb*. As illustrated in **Figure 7A**, carp TRIF-expressing plasmids significantly induced the luciferase activity of *ifn-1* and *nf-κb*, and this induction occurred in a dose-dependent manner in the *ifn-1* promoter activity assay. Moreover, we tested the effects of different carp TRIF mutants on *ifn-1* and *nf-κb* promoter luciferase activities. The results showed that carp TRIF-FL, TRIF-△N, and TRIF-△TIR had significantly increased the relative activity of *ifn-1* and *nf-κb*; however, TRIF-△C showed no influence compared with that of the control group **(** **Figure 7B** **)**. As shown in **Figure 7C**, TRIF-FL, TRIF-△N, TRIF-△TIR, and TRIF-△C proteins were strongly expressed in 293T cells at 48 h post-transfection. These results imply that carp TRIF relies on the C-terminal domain to activate the transcription factors.

### Carp TIR-Domain-Containing Adapter-Inducing Interferon-β (TRIF) Involved in the Antiviral Response *via* the TRAF6-TBK1 Axis

**Figure 8:**
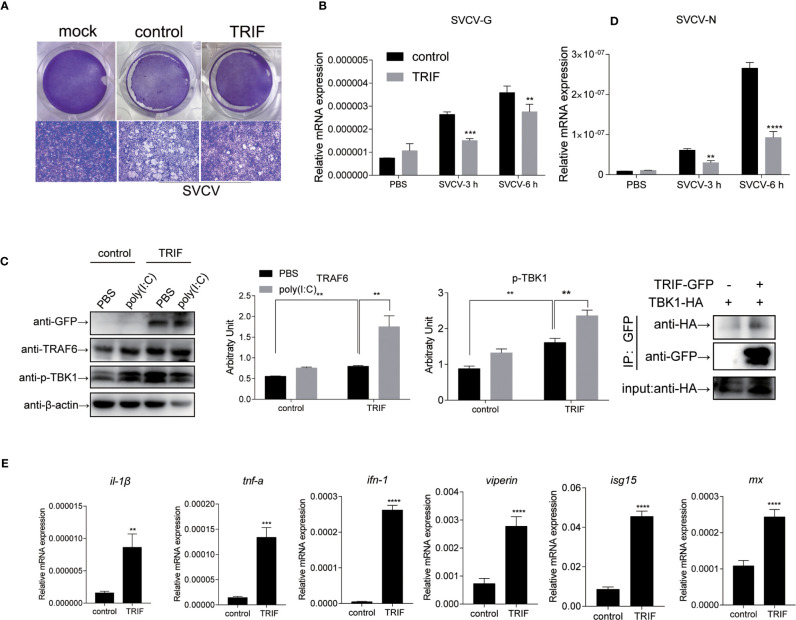
Carp TRIF involved in the antiviral response *via* the TRAF6-TBK1 axis. **(A)** EPC cells were seeded in 24-well plated overnight and transfected with pEGFP-N1 (control) or carp TRIF-EGFP. At 24 h post-transfection, the cells were infected with SVCV (MOI = 0.01) per well and incubated at 25°C for 24 h. This experiment was divided into two groups. **(A)** One group of cells stained with crystal violet for CPE. The cell monolayers were stained with crystal violet, and CPE was observed. **(B)** The second group of cells was used to extract the total RNA. The expression levels of SVCV-G and SVCV-N transcripts of SVCV by qRT-PCR analysis. **(C)** Cells were transiently transfected with above-mentioned plasmids in six-well plates. After 24 h of transfection, poly(I:C) stimulation was administered for 24 h, and then immunoblotted was conducted with the indicated antibodies. β-actin was used as a loading control. TRAF6/β-actin and p-TBK1/β-actin levels were quantified by the Image J software. **(D)** 293T cells were transfected with the indicated plasmids. At 48 h post-transfection, cell lysates were immunoprecipitated with anti-GFP, and immunoblotted analyzed with the indicated Abs. **(E)** EPC cells were transiently transfected with the indicated plasmids and harvested to quantify the relative expression levels of IFN-stimulated genes and proinflammatory genes at 6 h post SVCV infection. The results were calculated relative to the expression of EF-1α. All experiments were repeated at least three times with similar results. **P < 0.01, ***P < 0.001, ****P < 0.0001.

To further investigate the impact of carp TRIF on the antiviral activity, EPC cells were transfected with plasmid expressing carp TRIF and subjected to SVCV infection for 24 h. Crystal violet staining showed apparent CPE in the control cells, whereas the CPE was markedly reduced in TRIF-overexpressing cells after SVCV infection **(** **Figure 8A** **).** As shown in **Figure 8B**, the transcriptional levels of two SVCV genes (SVCV-G and SVCV-N) were significantly attenuated in carp TRIF-overexpressing cells at 3 and 6 h after SVCV infection, which suggest that carp TRIF can inhibit SVCV replication. To further explore the antiviral mechanism of carp TRIF, the expression of TRAF6 and TBK1, the downstream signaling of TRIF, was measured by WB analysis at 48 h post-transfection. As shown in **Figure 8C**, an enhanced Western blotting band was detected for TRAF6 and the phosphorylated form of TBK1 in carp TRIF-overexpressing cells. To further investigate the role of carp TRIF in the downstream signaling, EPC cells were stimulated with poly(I:C), in which poly(I:C) induced the expression of TRAF6 and phosphorylation of TBK1. The densitometric analysis of protein bands using the ImageJ software confirmed the results. Previous studies demonstrated that human TRIF forms a complex with TBK1 and TRAF6 (9). However, fish TRIF lacks the TRAF6 binding motif. Then, the interaction between carp TRIF and TBK1 was examined. Coimmunoprecipitation experiments indicated that carp TRIF was directly bound to TBK1 (**Figure 8D**). Next, the expression of IFN-stimulated genes and proinflammatory cytokines was detected in the carp TRIF-mediated immune response. After SVCV stimulation, the expression of *ifn*, *viperin*, *isg15*, *mx*, and proinflammatory cytokines such as *il-1β* and *tnf-α* were significantly upregulated in the carp TRIF-overexpressing group compared with the control **(** **Figure 8E** **)**. These results indicate that carp TRIF is involved in the antiviral response *via* the TRAF6-TBK1 axis.

### Carp TIR-Domain-Containing Adapter-Inducing Interferon-β (TRIF) Induces Apoptosis Through the Caspase-8 Axis

**Figure 9:**
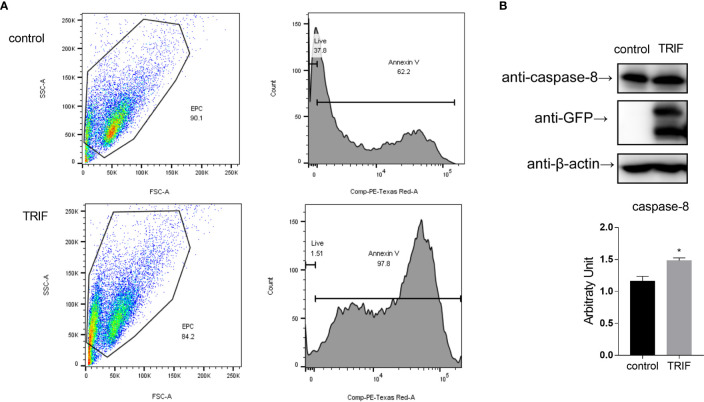
Carp TRIF induces apoptosis. **(A)** EPC cells in 24-well plates were transfected with pEGFP-N1 (control) or carp TRIF-EGFP. The early apoptosis cells were stained with Annexin V-mCherry. Flow cytometric analysis was undertaken to detect Annexin V in cells. The percent of apoptotic cells were determined using the FlowJo software. **(B)** 293T cells were transfected with pEGFP-N1 (control) or carp TRIF-EGFP. After transfection for 24 h, the cell lysates were collected. Western blot was performed with anti-caspase-8 Abs. The level of caspase-8 was quantified by densitometry analysis of the band intensity using the Image J software, and normalized to the β-actin. Data were shown as the mean ± SD of the relative ratios of the intensity (caspase-8/β-actin). All experiments were repeated at least three times. *P < 0.05.

Apoptosis is a host defense against pathogen invasion. To investigate whether carp TRIF can mediate apoptosis, EPC cells were transfected with carp TRIF and the early apoptosis was examined. The results demonstrated that the number of early apoptotic cells significantly increased in carp TRIF-overexpressing cells (97.8%) compared with the control group (62.2%) **(** **Figure 9A** **)**. To better define the mechanism by which carp TRIF initiates apoptosis, we examined the expression of caspase-8. As shown in **Figure 9B**, carp TRIF promoted caspase-8 activation. Hence, carp TRIF induces apoptosis through the caspase-8 axis.

## Discussion

TRIF is an essential adaptor protein required for innate immune responses mediated by TLRs (30). In teleost fish, several fish-specific TLRs, such as Tlr22 and Tlr19, can recruit TRIF and subsequently activate the TRIF-dependent pathway (20, 31), collectively implying that fish TRIF has an important role in the defensive immune responses. In the current study, the TRIF gene of common carp was cloned and characterized. Carp TRIF lacks the N-terminal and C-terminal proline-rich domains, which was reported in zebrafish, large yellow croaker, black carp, and orange spotted grouper (16, 18, 19, 21). Furthermore, multiple sequence alignment of the TIR domain among different species showed that carp TRIF displayed a typical TIR domain and had three highly conserved regions, Box 1, Box 2, and Box 3, which are conserved in mammalian TRIF and essential for TRIF- and MyD88-mediated signal transduction (6). In addition, phylogenetic analysis revealed that carp TRIF clustered with other fish TRIF and was highly similar to grass carp.

Tissue expression analysis showed that carp TRIF was constitutively expressed in several studied tissues of healthy common carp, with the highest expression levels detected in the spleen, indicating a possible role of TRIF in the immune defense system of common carp. However, the expression pattern was different in various species. Grass carp TRIF is highly expressed in the foregut and skin (17). Channel catfish TRIF has the highest expression in the ovary and spleen (15). In the amphioxus, transcripts of TRIF are strongly detected in the epithelial cells of the gut, skin, and gill (32). In zebrafish, a high TRIF transcript level is found in the liver (33). The differences in tissue distribution imply that TRIF performs divergent roles in various organs in different species. TLRs and their adaptors have a specific localization in the cells for their functions (34). In the current study, carp TRIF localized to a unique site near the nuclear membrane and was a Golgi apparatus-localized protein, which was consistent with demonstrations in other species, such as zebrafish (16) and orange-spotted grouper (18).

Bacteria are pathogenic agents of fish disease, and fish TRIF is reported to play important roles in the innate immune responses against bacterial pathogens. Channel catfish TRIF mRNA shows an enhanced expression in the head kidney and spleen after *Edwardsiella ictalurid* infection (15). The mRNA expressions of *TRIF* from orange-spotted grouper and mandarin fish are upregulated after LPS stimulation (18, 35). The transcription of amphioxus TRIF is upregulated after a challenge with gram-negative and gram-positive bacteria (32). Similarly, in the current study, the transcription of carp TRIF was upregulated in immune-related tissues after it was challenged with killed *A. hydrophila*, except in the liver. Additionally, fish TRIF is found to take part in the antiviral immune response. For example, the miiuy croaker TRIF contributes to IFN antiviral immunity following infection with Siniperca chuatsi rhabdovirus (SCRV) (36). In addition, TRIF expression was significantly upregulated in immune-related tissues in grass carp (17), large yellow croaker (34), zebrafish (16), and orange-spotted grouper (18) after poly(I:C) stimulation. Similarly, carp TRIF was upregulated after poly(I:C) stimulation *in vivo* and *in vitro*. These results indicate that fish TRIF exhibits extraordinarily broad roles in host antibacterial and antiviral innate immunity, which may be related to TLR recognition (20, 31).

To characterize the antiviral effects of carp TRIF, antiviral activity was investigated in carp TRIF-overexpressing cells. The results demonstrated that carp TRIF can inhibit the replication of SVCV *in vitro*. Similarly, in grass carp TRIF-overexpressing cells, the viral load and titer are significantly lower than those in the controls after GCRV challenge, and viral replication is obviously inhibited (17). Overexpression of grouper TRIF in grouper brain cells restrains the replication of red-spotted grouper nervous necrosis virus (RGNNV) (18). The possible mechanism of host defense against pathogens is inducing the expression of proinflammatory cytokines or interferon-stimulated genes. It is reported that mammalian TRIF can induce NF-κB and IFN-β (11). The activation of NF-κB in the TRIF-dependent pathway is verified to occur through the recruitment of TRAF6 and RIP1 by the TRAF6 binding motif and C-terminal RHIM domain, respectively (37–39). However, fish TRIF lacks the TRAF6 binding motif and C-terminal RHIM domain. Interestingly, carp TRIF significantly activated NF-κB, which was consistent with the findings in zebrafish, orange-spotted grouper, and large yellow croaker (16, 18, 34), implying the conserved function of fish TRIF in the NF-κB-mediating signaling cascade. Additionally, zebrafish TRIF is documented to associate with RIP1 but not TRAF6, and mutation of the TRIF RHIM domain disrupts NF-κB activation (16). Similarly, the absence of the C-terminus in carp TRIF suppressed the activation of NF-κB, indicating a fish TRIF-dependent NF-κB signaling *via* interaction with RIP1.

Mammalian TRIF is demonstrated to induce the production of IFN-β *via* the association of TRIF-TBK1 through the TBK1 binding motif (9). However, the TBK1 binding motif mentioned above is not conserved in fish TRIF. Intriguingly, carp TRIF could interact with TBK1, as found in zebrafish, indicating the conserved TRIF-TBK1 signaling cascade in teleost fish. Moreover, carp TRIF was capable of activating type I IFN, and a similar phenomenon was also observed in large yellow croaker (19), suggesting that type I IFN signaling pathways are involved in the TRIF-mediated immune response. Notably, it has been revealed that mutation of the C-terminal RHIM domain in common carp diminishes the activation of IFN, as shown in zebrafish (16), indicating that not only does the TBK1-mediated signaling cascade participate in TRIF-induced type I IFN production, but other molecules, such as RIP1, may also be involved in IFN activation. These results collectively suggest that fish TRIF may have different ways to induce IFN responses.

Apoptosis is a host defense against pathogen invasion that can be triggered by TLR ligands (40). Apoptosis occurs through the activation of members of the caspase family of cysteine proteases (41). Studies in mammals have demonstrated that protein-protein interactions resulting from the death domain (DD) are often involved in caspase activation (42). However, TRIF, which lacks DD, efficiently induces apoptosis (11). In this study, we found that carp TRIF overexpression induced apoptosis in EPC cells. Additionally, mammalian TRIF-induced apoptosis occurred through the activation of the FADD-caspase-8 axis (11). Similarly, carp TRIF overexpression enhanced the expression of caspase-8, suggesting that carp TRIF can induce apoptosis *via* the caspase-8 axis.

In summary, this study reported the identification and characterization of the TRIF gene from common carp. Carp TRIF participated in antibacterial and antiviral immune responses. In addition, overexpression of carp TRIF activated interferons and proinflammatory cytokines *via* the *ifn* and *nf-κb* pathways and inhibited SVCV replication in EPC cells. Furthermore, carp TRIF induced apoptosis through the activation of caspase-8. These results contribute to developing strategies for defense against virus infections in teleosts.

## Data Availability Statement

The datasets presented in this study can be found in online repositories. The names of the repository/repositories and accession number(s) can be found in the article/**Supplementary Material**.

## Ethics Statement

The animal study was reviewed and approved by the Animal Experimental Ethics Committee of Shandong Normal University.

## Author Contributions

SS conceived and designed the experiments. RL, XL, and MS performed the experiments and analyzed the data. YQ, HL, and GY participated in the discussion of the results. SS, RL, and GY designed the study and wrote the paper. All authors contributed to the article and approved the submitted version.

## Funding

This work was supported by the National Natural Science Foundation of China (32002419, 31972828) and the National Key R&D Program of China (2018YFD0900302-8).

## Conflict of Interest

The authors declare that the research was conducted in the absence of any commercial or financial relationships that could be construed as a potential conflict of interest.

## Publisher’s Note

All claims expressed in this article are solely those of the authors and do not necessarily represent those of their affiliated organizations, or those of the publisher, the editors and the reviewers. Any product that may be evaluated in this article, or claim that may be made by its manufacturer, is not guaranteed or endorsed by the publisher.

## Supplementary Material

Supplementary Figure 1 Nucleotide and deduced amino acid sequence of carp TRIF. The cDNA sequence and amino acid sequence of TRIF in Carp. Uppercase letters denote the coding region and lowercase letters show the UTR. The deduced amino acid sequence is shown below the coding regions and the polyadenylation signal attaaa is in bold font. The red box represents the TIR domain.

Supplementary Figure 2 The multiple alignment of TRIF across species. The sequences were aligned using the Clustal W method. The identical, conservative and similar substituted amino acid residues are indicated in (*), (: or .), respectively.
