# Supplementary figures and images for: Cyprinus carpio TRIF Participates in the Innate Immune Response by Inducing NF-κB and IFN Activation and Promoting Apoptosis
Source: Front Immunol. 2021 Aug 24;12:725150. doi: 10.3389/fimmu.2021.725150 (PMC8421551; doi:10.3389/fimmu.2021.725150)

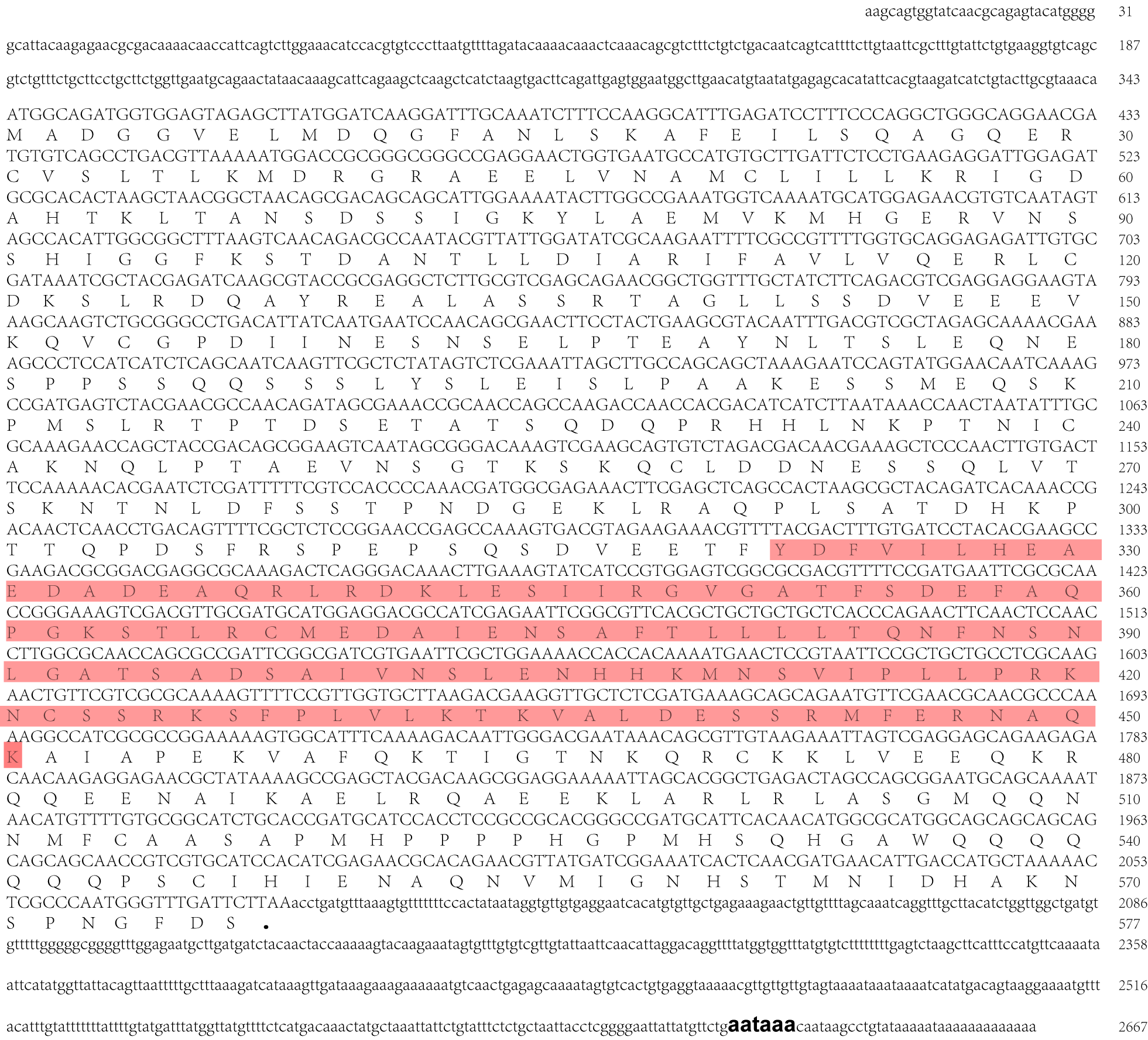

Supplement: Supplementary Figure 1 — Nucleotide and deduced amino acid sequence of carp TRIF. The cDNA sequence and amino acid sequence of TRIF in Carp. Uppercase letters denote the coding region and lowercase letters show the UTR. The deduced amino acid sequence is shown below the coding regions and the polyadenylation signal attaaa is in bold font. The red box represents the TIR domain. [file Image_1.tif]

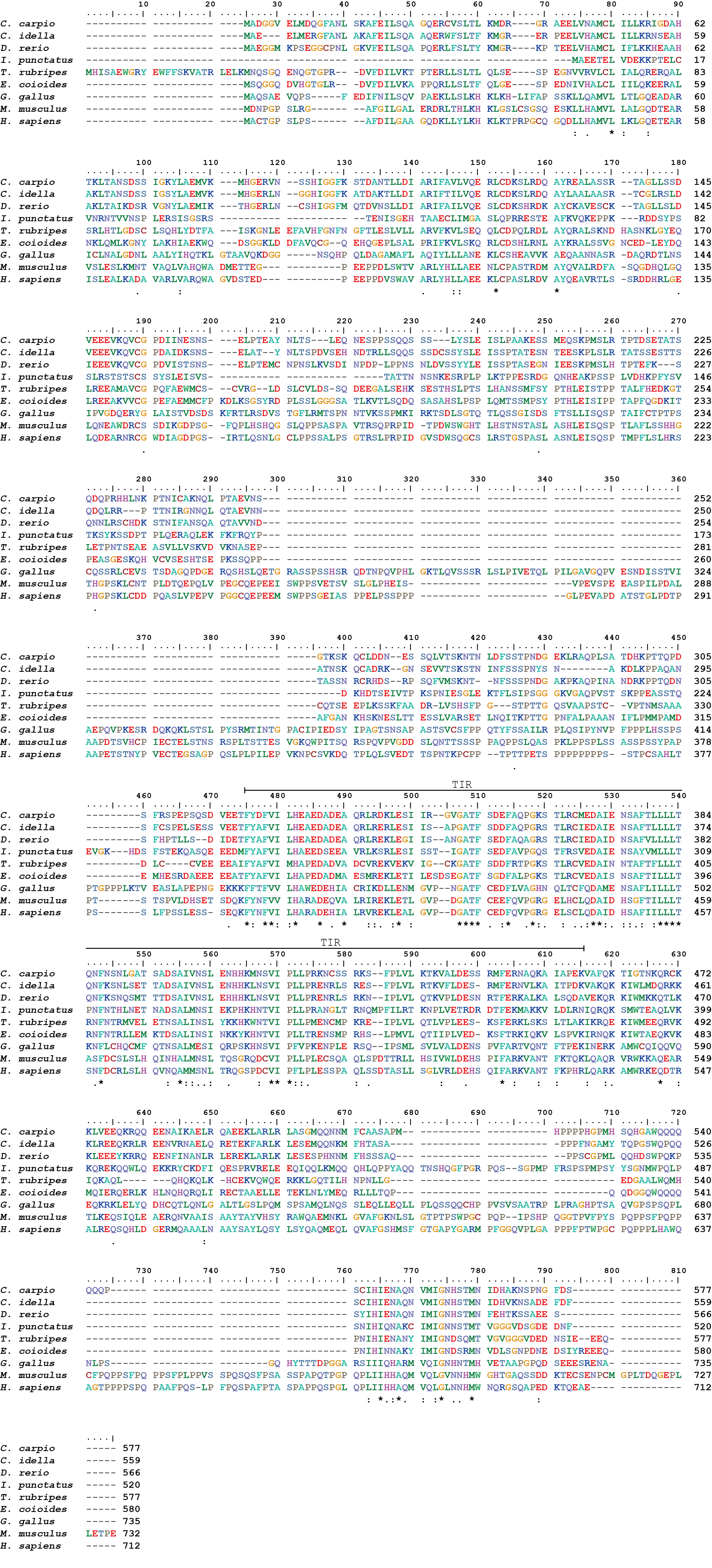

Supplement: Supplementary Figure 2 — The multiple alignment of TRIF across species. The sequences were aligned using the Clustal W method. The identical, conservative and similar substituted amino acid residues are indicated in (*), (: or .), respectively. [file Image_2.tif]
