# Supplementary material for: Cyprinus carpio TRIF Participates in the Innate Immune Response by Inducing NF-κB and IFN Activation and Promoting Apoptosis
Source: Front Immunol. 2021 Aug 24;12:725150. doi: 10.3389/fimmu.2021.725150 (PMC8421551; doi:10.3389/fimmu.2021.725150)
Supplement: Supplementary file 3 [file Table_1.docx]

Supplementary Table 1. Primer sequences used in this study.

| Primer name | Sequence (5’–3’) | Application |
| --- | --- | --- |
| Trif-F | GGTGCAGGAGAGATTGTGCGACAA | cDNA clone |
| Trif-R | TTCTGCGCGTTCTCAATGTGGATG | cDNA clone |
| Trif-5’outer | ACCAGCCGTTCTGCTCGACGCAAG | 5’ RACE |
| Trif-5’inner | GTTCTGCTCGACGCAAGAGCCTCG | 5’ RACE |
| Trif-3’outer | GCGCAACCGGGAAAGTCGACGTTG | 3’ RACE |
| Trif-3’inner | GCGCAACCAGCGCCGATTCGGCGA | 3’ RACE |
| Trif-HindIII-F | CCCAAGCTTATGGCAGATGGTGGAGTAGA | Plasmid construction |
| Trif-KpnI-R | CGGGGTACCGTAGAATCAAACCCATTGGGCGAG | Plasmid construction |
| Trif-△N-F-GFP | CAGATGGTGGAGTAGAGCTTATCAATG AATCCAACAGCGA | Plasmid construction |
| Trif-△N-R- GFP | TCGCTGTTGGATTCATTGATAAGCTCTA CTCCACCATCTG | Plasmid construction |
| Trif-△TIR-F- GFP | TAGAAGAAACGTTTTACGACGCCATCG CGCCGGAAAAAGT | Plasmid construction |
| Trif-△TIR-R- GFP | ACTTTTTCCGGCGCGATGGCGTCGTAA AACGTTTCTTCTA | Plasmid construction |
| Trif-△C-F- GFP | AACGCAACGCCCAAAAGGCCACGGTA CCGCGGGCCCGGGA | Plasmid construction |
| Trif-△C-R- GFP | TCCCGGGCCCGCGGTACCGTGGCCTTT TGGGCGTTGCGTT | Plasmid construction |
| rtTrif-F | GCTCACCCAGAACTTCAACTCCAA | Real-time PCR |
| rtTrif-R | GCGAGGTAGCAGCGGAATTACG | Real-time PCR |
| rtS11-F | CCGTGGGTGACATCGTTACA | Real-time PCR |
| rtS11-R | TCAGGACATTGAACCTCACTGTCT | Real-time PCR |
| EPC-*ifn-1*-F | ATGAAAACTCAAATGTGGACGTA | Real-time PCR |
| EPC-*ifn-1*-R | GATAGTTTCCACCCATTTCCTTAA | Real-time PCR |
| EPC-*viperin*-F | AGCGAGGCTTACGACTTCTG | Real-time PCR |
| EPC- *viperin*-R | GCACCAACTCTCCCAGAAAA | Real-time PCR |
| EPC-*mx*-F | GGCTGGAGCAGGTGTTGGTATC | Real-time PCR |
| EPC-*mx*-R | TCCACCAGGTCCGGCTTTGTTAA | Real-time PCR |
| EPC-*isg15*-F | ACAGTCGGTGAACTCAAGCAAGTC | Real-time PCR |
| EPC- *isg15*-R | CGTAACTGCTGAGGCTTCTGGAAT | Real-time PCR |
| EPC-*il-1β*-F | AAGCCGTGCTAATAAACCATCC | Real-time PCR |
| EPC-*il-1β*-R | ACCAGAATGACAACCTCCTGTTC | Real-time PCR |
| EPC-*tnf-a*-F | GTGATGGTGTCGAGGAGGAAG | Real-time PCR |
| EPC-*tnf-a*-F | TCTGAGACTTGTTGAGCGTGAA | Real-time PCR |
| EPC-EF1-α-F | AAGAGCGTTGAGAAGAAAG | Real-time PCR |
| EPC-EF1-α-F | GAGTGCCCAGGTTTAGAG | Real-time PCR |
