# Supplementary material for: Cyprinus carpio TRIF Participates in the Innate Immune Response by Inducing NF-κB and IFN Activation and Promoting Apoptosis
Source: Front Immunol. 2021 Aug 24;12:725150. doi: 10.3389/fimmu.2021.725150 (PMC8421551; doi:10.3389/fimmu.2021.725150)
Supplement: Supplementary file 4 [file Table_2.docx]

Supplementary Table 2. The GeneBank accession numbers of TRIF from other species

| Species | Protein | Accession number |
| --- | --- | --- |
| *Homo sapiens*  *Mus musculus*  *Gallus gallus*  *Danio rerio*  *Takifugu rubripes*  *Ictalurus punctatus*  *Ictalurus furcatus*  *Ctenopharyngodon idella*  *Epinephelus coioides* | TRIF  TRIF  TRIF  TRIF  TRIF  TRIF  TRIF  TRIF  TRIF | NP_891549.1  NP_778154.1  NP_001074975.1  NP_001038224  NP_001106665.1  NP_001187154.1  ABH10662.1  AGW25589.1  AEX01719.1 |
