# Supplementary material for: Cyprinus carpio TRIF Participates in the Innate Immune Response by Inducing NF-κB and IFN Activation and Promoting Apoptosis
Source: Front Immunol. 2021 Aug 24;12:725150. doi: 10.3389/fimmu.2021.725150 (PMC8421551; doi:10.3389/fimmu.2021.725150)
Supplement: Supplementary file 5 [file Table_3.docx]

Supplementary Table 3. Similarity of TRIF of carp and other species

| **Species** | **Identity** |
| --- | --- |
| *Homo sapiens*  *Mus musculus*  *Gallus gallus*  *Danio rerio*  *Takifugu rubripes*  *Ictalurus punctatus*  *Ictalurus furcatus*  *Ctenopharyngodon idella*  *Epinephelus coioides* | 24.8%  22.3%  23.6%  62.1%  30.5%  46.4%  31.4%  73.1%  32.4% |
